# The influence of stressful life events on procrastination among college students: multiple mediating roles of stress beliefs and core self-evaluations

**DOI:** 10.3389/fpsyg.2023.1104057

**Published:** 2023-05-12

**Authors:** Xiaole Ma, Zeheng Li, Furong Lu

**Affiliations:** School of Education Science, Shanxi University, Taiyuan, China

**Keywords:** stressful life events, procrastination, core self-evaluations, stress beliefs, college students

## Abstract

**Introduction:**

Few studies have documented the relationship between stressful life events and procrastination, which is a prevalent and troubling problem among college students. In this regard, the current study examined the association between stressful life events and procrastination through potential mediating effects of stress beliefs and core self-evaluations.

**Methods:**

A cross-sectional design was carried out and data were collected from a total of 794 Chinese college students with measures of stressful life events, core self-evaluations, stress beliefs, and procrastination.

**Results:**

Stressful life events was positively associated with procrastination in college students. Stress beliefs and core self-evaluations played multiple mediating roles in this relationship.

**Discussion:**

The study provided a novel perspective of finding the possible causes of procrastination in college students and highlighted the roles of stress beliefs and core self-evaluations.

## 1. Introduction

Procrastination, a maladaptive behavior that individuals delay an intended course of action regardless of possible negative consequences, is often characterized by voluntariness, avoidance and irrationality (Steel, 2007). Research interest in procrastination is increasing as it becomes more prevalent and exhibits high stability across time and context, as well as universality across cultural (Steel and Ferrari, 2013). Procrastination is also a challenging phenomenon among college students due to relatively independent yet complex external environment and immature mental development (Zhao et al., 2021). For college students, procrastination has been found to prevent success in academic learning, cause short-and long-term negative experience, and largely impair physical and mental health (Chen et al., 2020; Hong et al., 2021; Peixoto et al., 2021). While procrastination has been most investigated in the domain of academic behavior, it has also been transferred to work activities, as well as to various life domains such as health behaviors. However, knowledge regarding the processes that contribute to general procrastination of college students is not well established. Research needs to examine factors predicting procrastination among college students and the detailed mechanisms should be further elucidated for designing interventions.

### 1.1. Stressful lifer events and procrastination

According to ecological systems theory, individuals’ psychological development and behaviors are shaped by a variety of personal characteristics as well as environmental factors (Bronfenbrenner, 1977). A growing body of research has demonstrated that procrastination is shaped by personal factors such as conscientiousness (Gao et al., 2021), future time perspective (Liu and Feng, 2019), and self-control (Xu et al., 2021), as well as environmental factors such as parenting dimensions (Amani and Arbabi, 2020). As an environmental factor closely related to personal experience, stressful life events refer to the traumatic events and negative life changes in family, school and social life environment. In general, the evaluation of stressful life events relies on the objective situation, but is still affected by the lens through which the situation is perceived. The more stressful life events a person perceives, the worse they are likely to perform. It has been verified that stressful life events can result in psychological maladjustment, including individual well-being damage (Ouyang et al., 2021), depressive symptoms (Toyoshima et al., 2021), and externalizing problems (March-Llanes et al., 2017), like Internet gaming addiction (Sung et al., 2020), gambling (Wang et al., 2020), and even self-injury and suicide (Liu et al., 2019; Mo et al., 2019). Additionally, college students who have limited psychological resources are more likely to adopt negative coping methods such as withdrawal when exposed to more stressful life events (Li et al., 2009). Thus, perceived stressful life events may induce general negative implications for procrastination that are not limited to the academic domain. It is important to further explore whether stressful life events is associated with general procrastination in college students, and if so, what factors could be responsible for their association.

### 1.2. Stress beliefs as a mediator

Besides exerting a direct effect on procrastination, stressful life events may also affect procrastination through cognition, personality and other mediating factors. We first focused on a possibly crucial cognitive process in pathways from stressful life events to procrastination, namely one’s general attributes and expectations for stress, that is, stress beliefs (Crum et al., 2013). Stress beliefs are lay beliefs or lay theories about stress held by an individual, which can be formed by past experience of situations both empirically and vicariously, and influence how a person copes with stress (Kilby et al., 2020). According to theory of stress mindset, higher-level belief systems can explain interindividual differences in evaluations, reactions and results of stressful life events (Kilby et al., 2020). Previous findings have suggested that individuals with a positive stress beliefs showed higher cognitive flexibility and amplifying attentional bias to positive information (Crum et al., 2017). Again, negative stress beliefs have been linked to health- and performance-related problems, such as higher subjective stress appraisal, physiological stress responses and physical symptoms, and reduced academic performance in stressful situations (Fischer et al., 2016; Keech et al., 2018; Laferton et al., 2020). More importantly, the association between threat stress mindset and avoidance-motivated responses has been established (Jamieson et al., 2018). A recent study further indicated that exposure to stressful life events is related to avoidant coping strategies through the role of threat stress mindset (Chen and Qu, 2021). Based on prior theoretical and empirical grounds, we speculated that stress beliefs might also mediate the stressful life events–procrastination relationship.

### 1.3. Core self-evaluations as a mediator

Self-belief system model argues that risk factors exhibit an impact on adaptive consequences through self-system beliefs (Sandler, 2001), so core self-evaluations might also be a mediating variable worth considering. It is commonly accepted that core self-evaluations is a high-order, stable personality trait that manifests itself in at least four characteristics: self-esteem, generalized self-efficacy, neuroticism, and locus of control, representing a comprehensive appraisal of one’s own worth (Judge, 2009). Studies have proven that stressful life events can threaten positive self-schemas and increase the level of fatalism, thereby resulting in lower core self-evaluations (Orth and Luciano, 2015; Zuo et al., 2020). Furthermore, core self-evaluations reflect one’s response to self, others, environment and events, and play an important role in the process of adaptation to internal and external environment (Judge et al., 2003). For instance, individuals with low core self-evaluations are more likely to adopt inadequate coping styles like procrastination rather than proactive approach to solve problems (Geng et al., 2018). A longitudinal study further demonstrated that individuals with low self-esteem tend to avoid failure and maintain self-worth through procrastination, which is also known as self-handicapping (Yang et al., 2021). Thus, we proposed that core self-evaluations could also mediate the link between stressful life events and procrastination.

### 1.4. Stress beliefs and core self-evaluations as mediators

It is also logical to predict that stress beliefs and core self-evaluations have a sequential mediating role between stressful life events and procrastination. As a cognitive process, positive stress beliefs can function as a mediation mechanism in the prediction of family support on ones’ positive self-evaluations and psychological changes (Luu, 2022). Some researchers provided evidence that negative effect of beliefs about adversity, which was regarded as a type of cognitive heuristic that are related to appraisal or responses to stress, could be transmitted through self-esteem (Crum et al., 2017; Wang and Liu, 2022). Therefore, another aim of the present study is to confirm that stressful life events would result in negative stress beliefs, and then impair core self-evaluations, which increase the risk of procrastination.

### 1.5. Current study

Existing studies provide support for exploring the relationship between stressful life events and college students’ procrastination as well as the role of stress beliefs and core self-evaluations. Combined with the aforementioned ecological systems theory and self-system belief model, we constructed a multiple mediating model to test the following hypotheses: stressful life events relate with procrastination (H1) and stress beliefs and core self-evaluations mediate (H2).

## 2. Materials and methods

### 2.1. Participants and procedures

An anonymous cross-sectional sample were recruited through the network from several cities in China and a total of 876 college students completed the measurements. After deleting invalid questionnaires such as regular answers, self-report data were collected from 794 college students aged from 17 to 24 years (*M* ± SD = 19.81 ± 1.35 years) with an effective rate of 90.6%. The respondents include 306 males (38.54%; *M* = 19.65, SD = 1.59) and 488 females (61.46%; *M* = 19.90, SD = 1.17). Freshmen accounted for 37.5% (*n* = 298; male: *n* = 151), sophomores accounted for 30.9% (*n* = 245; male: *n* = 55), juniors accounted for 17.5% (*n* = 139; male: *n* = 47) and seniors accounted for 14.1% (*n* = 112; male: *n* = 53). The current study was approved by first author’s university ethics committee (No. SXULL2021082). The principle of confidentiality were explained and informed consent was provided by all the participants.

### 2.2. Measures

#### 2.2.1. Stressful life events

The 26-item version of Adolescent Self-rating Life Events Check-List was used to measure stressful life events (ASLEC; Xin and Yao, 2015). The scale described the following five aspects of life stress including interpersonal relationships (e.g., “I was misunderstood”), academic pressure (e.g., “I failed in the examination”), being punishment (e.g., “I was criticized and punished”), bereavement (e.g., “A family member/close friend died”), and health and adaptation problem (e.g., I was away from family). Participants were asked to report the degree to which each life event was applicable to their lives in the past six months using a six-point Likert scale with 0 representing “it never happened” and 1–5 representing “occurred and not stressful” to “occurred and extremely stressful,” respectively. The average score of all items was computed with a higher score indicating more perceived stressful experiences. In this study, the Cronbach’s *α* for the scale was 0.94.

#### 2.2.2. Stress beliefs

Stress beliefs was assessed using Beliefs about Stress Scale (BASS, Laferton et al., 2016; Pan et al., 2019). The scale consists of three subscales of negative stress beliefs (“being stressed makes me less resilient”), positive stress beliefs (“being stressed enables me to work in a more focused manner”), and control beliefs (“being stressed is something I am able to influence positively using my thoughts”). The questionnaire included 15 questions in total and seven of them were reversed scored. Participants were asked to rate the degree to which each item represented themselves on a five-point Likert scale from 1 (“completely disagree”) to 5 (“definitely agree”). The average score of all items was computed with a higher score indicating more positive beliefs on stress. The Cronbach’s *α* for this scale was 0.83.

#### 2.2.3. Core self-evaluations

The Core Self-Evaluations Scale (CSES, Judge et al., 2003) was used to measure the core self-evaluations. The scale included 10 items (e.g., “I am confident I get the success I deserve in life.”). A five-point Likert rating scale ranging from 1 (“strongly disagree”) to 5 (“strongly agree”) was employed to evaluate the score and six of them were reversed scored. The average score for all items was calculated and a higher score reflected higher level of self-evaluations. The Cronbach’s *α* for this scale was 0.85.

#### 2.2.4. Procrastination

The nine-item version of General Procrastination Scale (GPS-9, Sirois et al., 2019; Zhang et al., 2020) was used to measure procrastination in this study. Items included statements such as “I generally delay before starting work I have to do.” Questions were answered on a five-point Likert rating scale (1 = “strongly disagree”; 5 = “strongly agree”). The responses were averaged and three items were reversed scored so that a higher score reflected a greater tendency towards procrastination. The Cronbach’s *α* for this scale was 0.77.

### 2.3. Statistical methods

**Figure 1:**
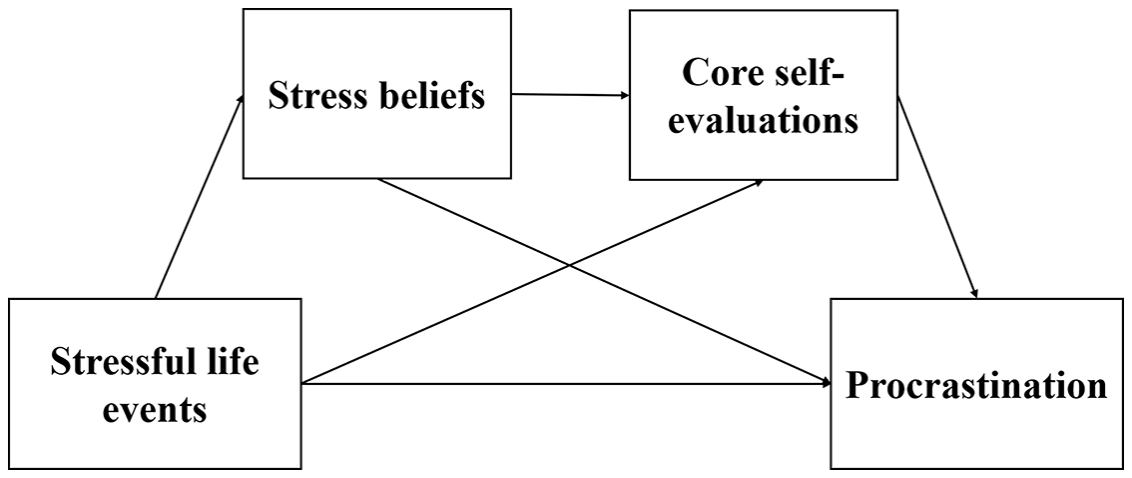
A hypothesized multiple mediating model.

In the present study, all statistical analyses were conducted using SPSS 24.0. Descriptive statistics and Pearson correlations were calculated for the main variables. The difference between males and females were assessed for significance using an independent sample *t*-test and Cohen’s *d* calculations. We first employed direction dependence analysis (DDA) in the PROCESS macro to assess the direction of the relationship among variables. DDA is a framework that is employed to confirm or disconfirm the ordering of a relationship by examining several properties of cross-sectional data (Wiedermann and Li, 2018). Stepwise linear regressions were then conducted to examine the independent associations between stressful life events and procrastination. Model 6 in the PROCESS macro (The model assumes that there are two mediation variables acting as sequential mediators) in SPSS developed by Hayes (2013) was adopted to examine the mediating effect. We used 5,000 bootstrapping samples to obtain the bootstrap confidence interval (CI) of parameter estimation and the effect was significant if the 95% CI does not include zero (see Figure 1).

## 3. Results

### 3.1. Preliminary analyses

As shown in Table 1, stressful life events was positively correlated with procrastination, negatively correlated with core self-evaluations and stress beliefs. Procrastination was also negatively correlated with stress beliefs and core self-evaluations. Stress beliefs was positively correlated with core self-evaluations. The independent sample *t*-test also showed that males perceived more stressful life events than females (*t* = 2.79, *p* < 0.01, Cohen’s *d* = 0.203). The DDA results were reported in supplementary materials. Briefly, the results at least partially supported pathway direction depicted in our model (Table 2, see details in Supplementary Tables S1–S3).

**Table 1:** Descriptive statistics and correlations among the variables (*N* = 794).

	Variable	1	2	3	4
1	Stressful life events	1			
2	Procrastination	0.22***	1		
3	Core self-evaluations	−0.31***	−0.46***	1	
4	Stress beliefs	−0.24***	−0.37***	0.59***	1
	*M*	1.28	2.72	3.30	3.22
	SD	0.79	0.62	0.61	0.49

^***^p < 0.001.

**Table 2:** Results of DDA analysis.

DDA properties	Target models					
	Stressful events → procrastination	Stressful events → stress beliefs	Stressful events → core evaluations	Belief in stress → procrastination	Belief in stress → core evaluations	Core evaluations → procrastination
**Variable distributions**						
Skewness diff (95% CI)	●			●		●
Kurtosis diff (95%CI)		○		●	●	
**Residuals distributions**						
Skewness diff (95% CI)	●	●	●			●
Kurtosis diff (95% CI)			●	●	●	
**Independence**						
Breusch–Pagan test			○		●	○
**DDA decision**	Target model	Target model (weak)	Target model (weak)	Target model	Target model	Target model (weak)

A solid dot indicates the hypothesized causal model (target model) is selected based on the test results. A circle indicates the reverse causal model is selected based on the test results.

### 3.2. The multiple mediation effect testing

**Figure 2:**
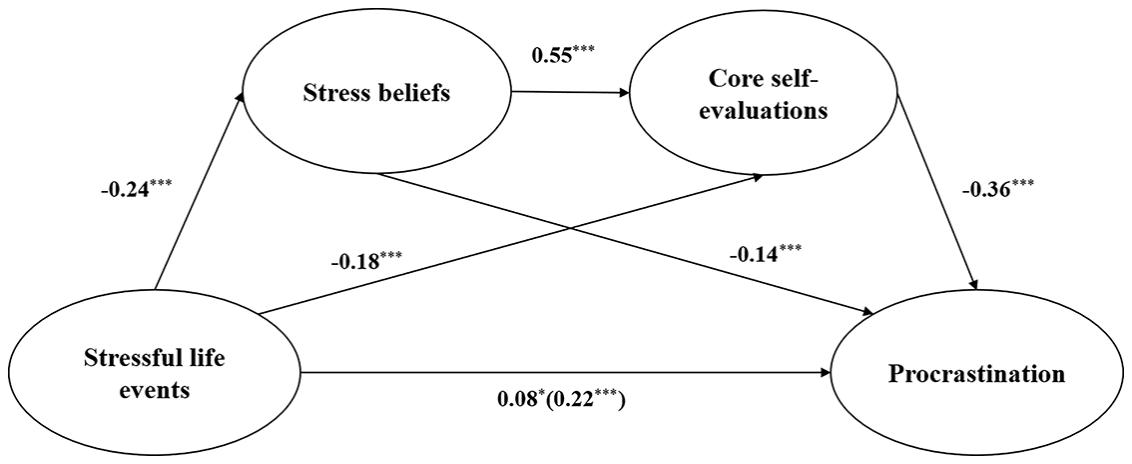
Mediating effect of stress beliefs and core self-evaluations on the relationship between stressful life events and procrastination. The total effect of stressful life events on procrastination was shown in parenthesis. ***p < 0.001; *p < 0.05.

As displayed in Table 3, stressful life events was positively associated with procrastination, *β* = 0.22, *p <* 0.001, 95% CI = [0.15, 0.30] (total effect, model 1). The results also showed that stressful life events had a significant positive association with stress beliefs, *β* = −0.24, *p <* 0.001, 95% CI = [−0.31, −0.17] (model 2). Model 3 showed that stressful life events (*β* = −0.18, *p <* 0.001, 95% CI = [−0.23, −0.12]) and stress beliefs (*β* = 0.55, *p <* 0.001, 95% CI = [0.48, 0.61]) were significantly associated with core self-evaluations, respectively. Besides, after adding the mediating variables to the model, the direct effect of stressful life events on procrastination was still significant (*β* = 0.08, *p <* 0.05, 95% CI = [0.02, 0.14]). Stress beliefs (*β* = −0.14, *p <* 0.001, 95% CI = [−0.22, −0.06]) and core self-evaluations (*β* = −0.36, *p <* 0.001, 95% CI = [−0.44, −0.27]) were positively associated with procrastination (model 4; Figure 2).

**Table 3:** Results of the multiple mediation analysis.

Predictors	Model 1 (procrastination)		Model 2 (stress beliefs)		Model 3 (core self-evaluations)		Model 4 (procrastination)	
	*β*	*t*	*β*	*t*	*β*	*t*	*β*	*t*
Stressful life events	0.22	6.43***	−0.24	−7.02***	−0.18	−6.05***	0.08	2.41*
Stress beliefs					0.55	18.90***	−0.14	−3.59***
Core self-evaluations							−0.36	−9.10***
*R^2^*	0.05		0.06		0.38		0.24	
*F*	41.37***		49.27***		238.63***		81.00***	

*p < 0.05, ***p < 0.001. Each variable in the model was standardized.

As shown in Table 4, analysis of the mediating effect showed the 95% CI of the various path coefficients did not include 0, which indicated that the total effects, direct effects, total mediating effects, and the indirect effects of the three mediation paths were all significant. The direct effect (effect size = 0.08) and total indirect effect (effect size = 0.14) accounted for 36.36% and 63.63% of the total effect (effect size = 0.22), respectively. Specifically, the indirect effect consists of three paths and accounted for 13.64%, 27.27% and 22.73% of the total effects, respectively. No significant differences were found when the effects of these paths were compared (*p* > 0.05).

**Table 4:** Bootstrap analysis of multiple mediation effects.

Path	Effect size	Boot SE	Boot LLCI	Boot ULCI	Relative effect
Total effects	0.22	0.03	0.15	0.30	
Direct effects	0.08	0.03	0.02	0.14	36.36%
Total indirect effects	0.14	0.02	0.11	0.18	63.63%
Stressful life events → stress beliefs → procrastination	0.03	0.01	0.01	0.06	13.64%
Stressful life events → core self-evaluations → procrastination	0.06	0.01	0.04	0.09	27.27%
Stressful life events → stress beliefs → core self-evaluations → procrastination	0.05	0.01	0.03	0.07	22.73%

Boot SE, Boot LLCI and Boot ULCI refer to the standard error, lower limit and upper limit of 95% CI of the effect estimated by percentile bootstrap method.

## 4. Discussion

To our knowledge, the present study is the first demonstration that individual cognitive factors (e.g., stress beliefs) and personal factors (e.g., core self-evaluations) can play a multiple role in the relationship between stressful life events and procrastination among college students, supporting the ecological systems theory and self-system belief model.

### 4.1. Relationship between stressful life events and procrastination

The current study explored the relationship between stressful life events and procrastination among college students. Hypothesis H1 was validated, that is, stressful life events was positively associated with procrastination among college students, which was consistent with previous studies indicating that stressful life events was associated with negative consequence and problematic behavior (Zuo et al., 2020; Geng and Lei, 2021).

According to the short-term mood regulation theory, stressful life events may lead to negative emotions so that individuals would give priority to short-term mood repair instead of long-term goal pursuit (Sirois and Pychyl, 2013). In addition, ego-depletion theoretical model posit that college students in a situation of high depletion caused by stressful life events prefer short-term satisfaction due to breakdown in self-control, which turn to a relatively stable, trait-like chronic tendency to delay (Gökalp et al., 2022). It reminds parents and schools to pay particular attention to college students experiencing more stressful life events so as to identify their difficulties and to improve their adjustment functions, which could eliminate the direct effect of stressful life events on procrastination. Some well-established interventions may also be applied in schools, concerning well-being and mindfulness, to reduce negative interpretation bias and favor positive attitudes (Gibb et al., 2022; Moè, 2022).

### 4.2. The multiple mediating effects

The results revealed that stress beliefs and core self-evaluations played multiple mediating effects in the relationship between stressful life events and procrastination, therefore, hypothesis H2 was validated. Stress belief had a mediating effect on the relationship between stressful life events and procrastination among university students. General frameworks regarding belief formation argue that beliefs are shaped by our personal context, upbringing, and lived and vicarious experiences (Kilby et al., 2020). Prior research, however, has shown no associations between stressful life events and stress beliefs in adolescents. These discrepancies could be due to the age difference that adolescence is a life stage of brain plasticity in which the beliefs about stress have not been shaped by the accumulation of stressful life events yet (Jiang et al., 2019). When stressful life events increases, individuals may repeatedly activate automatic negative thoughts, thereby tend to build up a belief that stress has a negative and threatening nature, which might lead to avoidance motivated responses (Chen and Qu, 2021). In this way, the intervention of stress beliefs in educational practice could be a method conducive to stress management, and change of irrational beliefs about stress can alleviate the adverse effects of stressful life events on procrastination (Keech et al., 2021). The results also indicated that core self-evaluations was a crucial explanatory mechanism in the association between stressful life events and procrastination, which supported hypothesis H3. Consistent with previous research, stressful life events made significant harmful impacts on core self-evaluations (Zuo et al., 2020). Exposure to stressful life events could make one develop the latent negative attributions and maladaptive schema, and generate cognitive biases that are associated with higher self-doubt and lower self-esteem (Han et al., 2018; Wong et al., 2021). According to the broaden-and-build theory of positive emotions, negative emotions caused by stressful life events would also hinder positive self-construction, thus leading to the reduction of core self-evaluations (Fredrickson, 2001).

This study also confirmed that core self-evaluations was negatively associated with procrastination. As a matter of fact, the damage of core self-evaluations leads to information processing bias, which inevitably affect individual coping process (Kammeyer-Mueller et al., 2009). Generally, lower self-esteem is associated with higher self-handicapping, which shares much in common with procrastination in terms of individual’s emotions, thoughts, reasons, and motives (Barutçu Yıldırım and Demir, 2020). The temporal motivation theory also posited that individuals with low core self-evaluations tend to underestimate their coping ability and have lower confidence in obtaining a desired reward or outcome, and tend to put things off (Steel, 2007; Zhang et al., 2019). This study further support the self-belief system model, namely the external risk factors (i.e., stressful life events) influence the adaptive consequences (i.e., procrastination) through individual self-belief system (i.e., core self-evaluations; Sandler, 2001). College students should improve their capacity to resist stressful life events, and adjust their non-adaptive self-cognition in difficulties and setbacks, so as to reduce procrastination. Meanwhile, schools can attach importance to psychological intervention for college students with low core self-evaluations to help them form a positive self-evaluation and obtain knowledge of self-leadership strategies, which may also be an effective way to reduce procrastination (Wang et al., 2021).

Stressful life events could be also associated with procrastination through a sequential mediating effect involving stress beliefs and core self-evaluations. These results provide a specific psychological elucidation for how stressful life events affect procrastination from both cognitive and personal perspective (Zuo et al., 2020). When college students are faced with amounts of stressful life events, they tend to hold negative stress beliefs that stress is threatening, negative and uncontrollable, thus leading to a reduction in the senses of self-evaluations, which make individuals prone to adopt a passive attitude and lead to an increased risk of procrastination ultimately (Chen and Qu, 2021). The results further extend the existing frameworks and verified a multiple path to procrastination.

### 4.3. Limitations and further directions

Despite the aforesaid implications, several limitations can be addressed in future research. First, given the cross-sectional design used in this study, the possibility of drawing causal conclusions was limited. Thus, a longitudinal approach to test the conclusion and effective intervention methods should be adopted to provide a deeper understanding. Second, college students volunteered to participate in the study online, which may be influenced by personality of participants and weaken the representativeness of the sample. Third, this study only surveyed Chinese college students, which poses a limitation in the generalizability of the present results to other cultures. Studies showed that people from different cultural backgrounds react differently to stress in respect of stress appraisal and support seeking (Tweed et al., 2004). At the same time, stress beliefs are correlated with optimism, which have distinctive cultural characteristics. It is reasonable to believe that culture affects the outcome of stress beliefs, and that the close relationship between stress beliefs and core self-evaluations may be affected by environmental and cultural factors (Jiang et al., 2014). Therefore, future studies may examine the model of this study by collecting data from different cultural background groups. Finally, the self-reported measures used in this study may not explain the problem in terms of objectivity and may be subject to social desirability response bias. Data was then collected anonymously to reduce this risk. Future research could benefit from repeated measurements, or non-self-reported measurements, as well as experiments with objective indicators and the combination of multiple measures.

## 5. Conclusion

In conclusion, this study identified the associations between stressful life events, stress beliefs, core self-evaluations, and procrastination. Specifically, stressful life events was directly associated with procrastination, or indirectly via the parallel mediating effects of stress beliefs and core self-evaluation, or via the sequential mediating effects of stress beliefs and core self-evaluations. The present study contributes to an understanding of mechanisms through which exposure to stressful life events predicts procrastination among college students and has certain theoretical and practical implications to understand the intervention of procrastination.

## Data availability statement

The raw data supporting the conclusions of this article will be made available by the authors, without undue reservation.

## Ethics statement

The studies involving human participants were reviewed and approved by Shanxi University. The patients/participants provided their written informed consent to participate in this study.

## Author contributions

XM conceived the experimental design and drafted the manuscript. XM and ZL contributed to the data collection and preparation of the statistical analyses. FL was mainly responsible for the language polishing and proofreading. All authors contributed to the article and approved the submitted version.

## Conflict of interest

The authors declare that the research was conducted in the absence of any commercial or financial relationships that could be construed as a potential conflict of interest.

## Publisher’s note

All claims expressed in this article are solely those of the authors and do not necessarily represent those of their affiliated organizations, or those of the publisher, the editors and the reviewers. Any product that may be evaluated in this article, or claim that may be made by its manufacturer, is not guaranteed or endorsed by the publisher.
