# Supplementary material for: The influence of stressful life events on procrastination among college students: multiple mediating roles of stress beliefs and core self-evaluations
Source: Front Psychol. 2023 May 12;14:1104057. doi: 10.3389/fpsyg.2023.1104057 (PMC10213235; doi:10.3389/fpsyg.2023.1104057)
Supplement: Supplementary file 1 [file Data_Sheet_1.PDF]

Supplementary materials

We provide a detailed description of the results of our Direction of Dependence Analyses (DDA; Wiedermann & Li, 2018) for "The influence of stressful life events on procrastination among college students: Multiple mediating roles of stress beliefs and core self-evaluations".

Given the cross-sectional design used in this study, the possibility of drawing causal conclusions was limited. To explore alternate causality and quantify the evidence in favor of each causal pathway, we implemented DDA. This statistical technique aims to gain insight into the data generating mechanism of cross-sectional data by looking at the distributional characteristics of the variables involved. Specifically, it has been shown that the following properties can convey information about causality: (1) the distribution of hypothesized predictor and outcomes variables, (2) the distribution of residuals of competing causal models, and (3) independence of predictors and errors of competing models (Wiedermann et al., 2015). For the first criterion, statistical tests investigates null hypotheses related to normality of the observed variables and the tenet is that the distribution of outcome variables will be closer to normality than the distribution of predictor variables in a correctly specified causal model. For the second criterion, a series of statistical tests in DDA examines null hypotheses related to normality of the residuals of two competing causal models and the tenet is that the distribution of residuals will also be closer to normality in a correctly specified causal model (compared to a mis-specified model). For the third criterion, statistical tests investigates whether the assumption of independence (between predictor and residuals) is confirmed for a given causal sequence and disconfirmed for the competing causal sequence and the tenet is that predictors and residuals are more likely to be independent in a correctly specified causal model (compared to a mis-specified model).

In line with DDA principles, we thus fitted and compared the distributional characteristics of our hypothesized causal chains and the reverse causal chains. The results of

these analyses depicted in Tables 1, 2, and 3, provided at least partial support for the relationship in our specified causal model.

In the first step of our DDA analyses (Table S1), we assessed indicators of normality (i.e., skewness and kurtosis) for the distributions of our focal variables. Skewness or kurtosis was significantly different for hypothesized outcome and predictor, which partially supported several pathways in hypothesized model (stressful events -> procrastination; belief in stress -> procrastination; belief in stress ->core evaluations; core evaluations -> procrastination). The results of these tests partially supported the reverse causal model (stressful events ->stress beliefs).

In the second step of our DDA analyses (Table S2), we investigated whether skewness and kurtosis of residuals are significantly different for hypothesized and reverse causal model, and results provided partial support for all pathways in hypothesized model, and no evidence in favor of the reverse model.

In the third step of our DDA analyses (Table S3), we investigated whether predictors and errors were indeed independent in hypothesized causal model and reverse causal model. Results partially supported several pathways in hypothesized model (belief in stress -> core evaluations). The results of these tests partially supported the reverse causal model in several pathways (stressful events >core evaluations; core evaluations-> procrastination) and provided support for both causal models in a pathway (stressful events->procrastination).

Wiedermann, W., Hagmann, M., & von Eye, A. (2015). Significance tests to determine the direction of effects in linear regression models. *British Journal of Mathematical and Statistical Psychology, 68*(1), 116–141.https://doi.org/10.1111/bmsp.12037

Wiedermann, W., & Li, X. (2018). Direction dependence analysis: A framework to test the direction of effects in linear models with an implementation in SPSS. *Behavior Research Methods, 50,* 1581-1601. https://doi.org/10.3758/s13428-018-1031-x

Table S1

Output of DDA Dependent Variable Normality Hypothesis Tests

Test	A/B~*N*?				A ≠ B?			
	Skew		*Excess Kurtosis*		*Skew*		*Excess Kurtosis*	
	A	B	A	B	Δ	95% BootCI	Δ	95% BootCI
A: stressful events B: procrastination	**0.64*** **	**-0.12**	0.21	0.11	**0.76**	**[0.29,0.71]**	0.10	[-0.50,0.69]
A: stressful events B: stress beliefs	0.64***	0.43***	0.21	1.09***	0.21	[-0.10,0.51]	*-0.88*	*[-1.58,-0.13]*
A: stressful events B: core evaluations.	0.64***	0.44***	0.21	-0.08	0.20	[-0.01,0.41]	0.29	[-0.27,0.88]
A: belief in stress. B: procrastination.	**0.43*** **	**-0.12**	**1.09*** **	**0.11**	**0.55**	**[0.06,0.57]**	**0.98**	**[0.40,1.51]**
A: belief in stress. B: core evaluations.	0.43***	0.44***	**1.09*** **	**-0.08**	-0.01	[-0.25,0.21]	**1.17**	**[0.65,1.62]**
A: core evaluations B: procrastination.	**0.44*** **	**-0.12**	-0.08	0.11	**0.56**	**[0.13,0.48]**	-0.19	[-0.50,0.16]

Note. A = hypothesized predictor. B = hypothesized outcome. A/B~*N*? refers to the skewness and kurtosis tests investigating whether normality holds for the variable. A ≠ B? refers to tests investigating whether skewness and kurtosis are significantly different for hypothesized outcome and predictor (significant positive difference reflects support for hypothesized model). Results of tests in line with the hypothesized causal model are printed in bold, Results of tests in line with the reverse causal model are printed in italic. ***: $p < .001$.

Table S2

Output of DDA Competing Models Normality of Residuals Hypothesis Tests

Test	A/B~*N*?				A ≠ B?							
	Skew		*Excess Kurtosis*		*Skew*				*Excess Kurtosis*			
	A	B	A	B	Δ	*z*	*p*	95% BootCI	Δ	*z*	*p*	95% BootCI
A: stressful events->procrastination.	**-0.09**	**0.64*****	0.05	0.25	**0.55**	***-5.56***	**<0.001**	**[0.34,0.74]**	0.20	-0.72	0.47	[-0.33,0.77]
A: stressful events ->stress beliefs	0.34***	0.69***	0.87***	0.39*	**0.36**	**-2.52**	**0.01**	**[0.05,0.65]**	-0.49	1.22	0.22	[-1.24,0.35]
A: stressful events -> core evaluations.	0.38***	0.66***	-0.22	0.29	**0.28**	**-1.98**	**0.05**	**[0.08,0.48]**	**0.06**	**-2.09**	**0.04**	**[0.02,1.00]**
A: belief in stress. ->procrastination.	0.19*	0.26**	**0.01**	**0.98*****	0.07	-0.59	0.56	[-0.22,0.34]	**0.97**	**-2.76**	**0.006**	**[0.36,1.54]**
A: belief in stress. ->core evaluations.	0.13	-0.11	0.71**	1.42***	-0.02	1.86	0.06	[-0.17,0.21]	**0.71**	**-1.47**	**0.14**	**[0.05, 1.41]**
A: core evaluations ->procrastination.	**0.10**	**0.36*****	0.19	0.14	**0.26**	**-2.18**	**0.03**	**[0.05,0.44]**	-0.05	0.18	0.86	[-0.47,0.36]

Note. A = hypothesized causal chain. B = reverse causal chain. *A/B ~ N?* refers to the skewness and kurtosis tests investigating whether normality holds for the residuals of the respective model. A ≠ B? refers to tests investigating whether skewness and kurtosis of residuals are significantly different for hypothesized and reverse causal model (significant positive difference reflects support for hypothesized model). Results of tests in line with the hypothesized causal model are printed in bold, results of tests in line with the reverse causal model are printed in italic. **: $p < .01$. ***: $p < .001$.

Table S3

Output of DDA Competing Models Independence Hypothesis Tests

	A ⊥ ε?		*B ⊥ ε?*	
	χ^2 *Standard*	*BP*	χ^2 *Standard*	*BP*
A: stressful events->procrastination.	0.78	0.77	2.17	1.94
A: stressful events-> stress beliefs	9.39**	6.53*	0.82*	0.68
A: stressful events >core evaluations.	4.48*	5.04*	*3.73*	*3.26*
A: belief in stress ->procrastination.	14.13***	14.03***	3.81**	2.56*
A: belief in stress -> core evaluations.	**0.30**	**0.22**	12.56***	7.34**
A: core evaluations-> procrastination.	5.58**	5.11**	*2.07*	*1.94*

Note. A = hypothesized causal chain. B = reverse causal chain. BP = Breusch-Pagan test. *A ⊥ ε?* refers to tests investigating whether predictors and errors are likely to be independent in hypothesized causal model. B *⊥ ε?* refers to tests investigating whether predictors and errors are likely to be independent in reverse causal model. Results of tests in line with the hypothesized causal model are printed in bold, results of tests in line with the reverse causal model are printed in italic. *: $p < .05$. ***: $p < .01$. ***: $p < .01$.
